# Home-Care Workers’ Representations of Their Role and Competences: A Diaphanous Profession

**DOI:** 10.3389/fpsyg.2020.581399

**Published:** 2020-12-10

**Authors:** Diletta Gazzaroli, Chiara D’Angelo, Chiara Corvino

**Affiliations:** Department of Psychology, Università Cattolica del Sacro Cuore, Milan, Italy

**Keywords:** elderly, care, competences, role representation, home-care workers, invisible welfare

## Abstract

Because of the gradual aging of the population, hospital facilities for socio-sanitary care of the elderly are quite scarce relative to the very high number of elderly people present in the country. This has pushed a high number of families to privately hire home-care workers. The scientific literature gives a picture of the psycho-physical risks that this type of profession is exposed to; however, there is still a need for a more systemic reflection with regard to representations about their role and competences. The aim of the present study is to outline the representations of the role and the skills it requires from home-care workers’ point of view. We reconstructed how home-care workers perceive and define the profession, and understand the necessary skills required from their point of view. Our results show that the professional profile of home-care workers still remains poorly defined and that professionals themselves struggle to find value and recognition, and to articulate what the skill set they develop is formed of.

## Introduction

Recently, the European Union estimated that the relative size of the elderly population in its member countries is destined to keep growing.

In parallel with the gradual aging of the population, professions dedicated to the care of the elderly will see stronger demand in the labor market, as they are crucial to handling this challenge (Hall and Coyte, 2001; McClimont et al., 2004; Broese van Groenou et al., 2006; Stone and Dawson, 2008; Gozzoli and Frascaroli, 2012; Scaratti et al., 2014, 2017; Bjerregaard et al., 2015; Gozzoli et al., 2018). Whereas in the past, care of the elderly was mainly the responsibility of relatives–specifically the women of the family–nowadays there is a greater presence of services and specific professions that are responsible for the socio-sanitary needs of the elderly (Ambrosini, 2013).

The progressive increase in the size of the elderly population will therefore be of particular and crucial interest for all countries in the EU, and this is especially true for Italy. Thus, if we look at our national context, Italy will be particularly affected by this phenomenon, as its elderly population is larger than that of other EU countries.

The Italian social welfare system has quite limited hospital facilities for socio-sanitary care of the elderly, compared to the very high number of elderly people present in the country. Among the regular forms of elderly care, despite the fact that there are different systems and governance depending on the region, care of the elderly can also be handled on a regional level by non-profit services such as social cooperatives that receive funding from municipalities and grant a series of regional services to the elderly (Scrinzi, 2019).

Demand, however, is much higher than supply, and this has pushed a high number of families who could not make use of informal care networks (Toniolo Piva, 2002) to privately hire home-care workers^1^ (Pasquinelli and Rusmini, 2013; Scrinzi, 2019).

Indeed, the lack of national resources to economically support families with elderly relatives who need care and the ever-increasing use of the so-called “Invisible Welfare” renders the private market for home-care workers the main means of welfare management for the Italian elderly, both via formal and informal arrangements (Ambrosini, 2013; Pasquinelli and Rusmini, 2013). Within the formal private market there are many services that perform a matchmaking role between demand and supply (Scrinzi, 2019); however, a common phenomenon is the undeclared, informal labor of millions of workers, mostly women, primarily migrants, who do not have any sort of labor protection (Sardadvar et al., 2012).

Nevertheless, to complement the picture we should bear in mind that this profession presents a work profile that is still not very well defined on a formal and training front. This becomes particularly relevant in the Italian context.

In Italy we can see an attempt, which is still in its initial stages, to define specific training pathways for home-care workers that, however, do not seem to be mandatory qualifying steps for the profession. Many workers, in fact, carry out this profession without taking part in such training. Where present–as, for instance, in the region of Lombardy–the training programs give a rather vague description of the profile of the home-care worker.

1. The family assistant carries out caregiving activities for people with different levels of psycho-physical self-sufficiency (elderly, sick, disabled), including in support of family members, contributing to the maintenance of the autonomy and well-being of the assisted person.

Particularly

– Assists the person from a domestic and hygienic point of view.
– Supports the psycho-physical well-being of the assisted person.
– Intervenes to support the maintenance and restoring of physical and psychological autonomy of the assisted person, reducing the risk of isolation.

(Region of Lombardy)

INAPP, (2020) (*Istituto nazionale per l’analisi delle politiche pubbliche*; National Institute for the Analysis of Public Policies)^2^, in collaboration with the Ministry of Labor and of Social Politics, created a system that observes and monitors the profession and its requirements.

For the home-care worker profession, which covers the group of people responsible for personal assistance, a series of competences and knowledge that should been acquired to be able to optimally carry out the profession have been outlined.

These competences, however, are not unique to the home-care worker, but give a wide picture that covers roles and duties of other professions as well, such as: social worker for domestic care, social security operator, worker responsible for assistance in dormitories, nursing aide for domestic care, animator in residences for the elderly, home attendant, assistant and chaperone for the disabled in institutions, family assistant, socio-sanitary assistant, socio-sanitary assistant with an educational role, and chaperone for an invalid person.

The presence of profiles similar to that of the home-care worker tells us about an extreme fragmentation of professions related to care, which, consequently, risks disseminating a picture of general competences that are ambiguous and that don’t involve psycho-relational aspects.

In this regard, INAPP mentions a wide range of desired skills for workers in the personal assistance field, including active listening, understanding of others, service orientation, time management, adaptability, ability to resolve complex problems, critical thinking, ability to analyze and resolve unpredictable problems, and decision-making–skills that, however, lack sophistication with respect to the psycho-emotional implications of home-care work.

### Home-Care Workers’ Attributes

The home-care worker profession has some peculiarities that are unique within the labor market.

Firstly, in contrast to other professional relationships, this profession presents some atypical interpersonal dynamics. Generally, the employer is in a superior hierarchical position, which leads to an imbalance of power toward the employee. Although in some cases this imbalance can be a fatiguing and frustrating condition, it also guarantees a clarity of boundaries between roles and duties. In contrast, in the case of a home-care worker, this hierarchical relationship–which has been studied by various authors across the years–is extremely controversial if not ambiguous, as well as hard to define (Baldassar et al., 2017).

Triandafyllidou and Marchetti’s (2015) report notes that, for example, employers–generally the relatives of the assisted–expect the employee to perform beyond the mere material care of the assisted, to provide for the overall well-being of the elderly. The employer therefore pays the employee in the expectation that the home-care worker will work not only by delivering a service but that he/she will also take care of–and therefore also show affection and love to–the no longer self-sufficient parent. The resulting emotional enmeshment is quite delicate. However, often, at the start of a work relationship, this dimension stays implicit, and during the working contract discussion there is no moment when the psychological contract between the parties is discussed. As a consequence of this, very often the professional contract is drafted only in terms of the performance component, neglecting the relational one almost entirely.

Uttal and Tuominen (1999, p. 776) also highlighted the contradictory nature of this profession, underlining how the intrinsic inequity in the employer-employee relationship is also combined with the fact that the job is emotionally intense for both the workers, who (often) find themselves in oppressive work conditions, and the employers, who in many cases have to process the sense of guilt and frustration.

Shifting the focus on the relationship between the professional and the assisted, there are other emerging forms of vulnerability that occur if the home-care worker does not receive appropriate training. The dynamic of a domestic worker being responsible for an elder is characterized by a series of specific difficulties caused by the long periods spent by the elder in solitude. Home-care assistance can be emotionally stressful, as the workers perceive that they are the only person responsible for their client. Moreover, the feeling of closeness with the client is accompanied by the need to keep one’s space and maintain psychological boundaries in order to protect oneself from the emotional charge that prolonged contact of this nature implies. Moreover, these types of workers find themselves facing themes of illness and death, and this can make them particularly vulnerable from a psycho-emotional point of view (Sardadvar et al., 2012).

There is therefore a strong emotional and relational component in this occupation, accompanied by atypical working conditions that can assume familial connotations. In fact, it is not rare for the employee to be considered part of the family over the long term (Näre, 2011). It is important to underline here that although it can seem to be a protective factor, perceiving the home-care worker as part of the family can make it even harder to consider him/her a professional and, consequently, recognize and maintain reciprocal safeguarding boundaries.

It is important to highlight that all these observations are even more significant for home-care workers who live and sleep with an elderly/disabled person. Thus, the level of emotional involvement of a home-care worker living with the elderly/disabled is higher than that experienced by those not living under the same roof.

Studies in the Italian context have mainly focused on socio-economic issues related to elderly care-work policies and trends (Glucksmann and Lyon, 2006; Lyon, 2006; Elrick and Lewandowska, 2008; Di Rosa et al., 2012), as well as gender and ethnicity issues related to care (Bettio et al., 2006; Näre, 2013; Ranci et al., 2019; Scrinzi, 2019).

There are also studies that analyze the emotional impact of the job from the perspectives of the workers (Degiuli, 2007). Within this framework, the Italian literature lacks studies on representations surrounding this professional role, especially from the point of view of home-care workers.

On a practical level, home-care workers are asked to carry out personal assistance duties that are relatively simple, for example bathing, dressing, or grooming, and light household tasks that do not require specific training (Sims-Gould and Martin-Matthews, 2010). On the other hand, relational and emotional challenges that these professions face highlight, in contrast, the need to adequately train these workers, especially in terms of their psycho-relational skills. The private market requires the home-care worker to have a series of soft skills that are quite vague and generally linked to the care and “mothering” of the assisted, without getting into more detail. All these skills that the home-care worker should develop–not only to take care of the elder but also to take care of their own well-being–remain completely absent and unexplored.

In sum, despite the fact that the future trend predicts a consistent increase in the demand for professions specialized in the care of the elderly, there is still a need for a more systemic reflection.

Starting with the definition of Dadich and Doloswala (2018), we refer to the role and competences as fundamental components of the professional identity. The exploration of such dimensions serves the systemic reflection on this profession.

As reported by Heldal et al. (2019), caregiving professions are less codified and standardized if compared to “hard” jobs, such as doctors. This is because they are generally characterized by implicit and tacit practices that are difficult to describe. This characteristic affects the social recognition of these jobs by third parties since it is hard to explicate the professional expertise at a societal level.

Specifically, in light of previous qualitative work on high-risk professions (D’Angelo et al., 2018; Gozzoli et al., 2018), we believe it is important to deepen role representations and specific competences for home-care workers, from their own point of view.

## Materials and Methods

### Aims and Scope

The aim of the present study is to outline with more clarity the profession of the home-care worker from their perspective. To fulfill this goal, the study seeks to:

(1) Explore and reconstruct how home-care workers perceive and define their professional role;
(2) Explore their representations about the necessary skills required for practising this job by analyzing stories of caregiving and caretaking practices (daily functions and challenges).

### Procedure

The participants were contacted through a network of a non-profit organization that operates in the Lombardy region to promote the integration of foreign citizens in Italy. The recruitment was carried out alongside the non-profit organization’s staff. In this phase, given the delicate topic of the research and possible resistance, it was deemed important to introduce the topic of the study to the possible participants through “familiar” figures who were considered “trustworthy” by the home-care workers. Explanations of the study’s procedure, the written consent form and the interviews, on the other hand, were conducted by the research team.

The conducting of the interviews showed us the difficulty of the home-care workers in explaining the skills required for the profession. This effort was common to all the interviews and led us to outline the repertoire of skills by using a process of *a posteriori* induction.

In order to do this, the thematic areas related to caregiving actions and the challenges with which the interviewees interface in their daily work were analyzed. Starting from these, the researchers reconstructed in an inductive way, from the particular to the generic, the skill set of the profession.

Since the skill set didn’t directly emerge from data, it will be presented in the discussion in the results section.

### Sample

It was decided to distribute the home-care workers (*n* = 30), all of whom were women, evenly across three main criteria:

– Nationality–to guarantee equal representation between Eastern Europe and South America, which were the two macro-areas that the homeworkers came from;
– Years of occupation–to guarantee an equal distribution between those who had experience of no more than 5 years, between 5 and 10 years, and more than 10 years;
– Past experience–to guarantee an equal distribution between those who had “positively” perceived experiences and those who had “negatively” perceived experiences.

Table 1 below presents the characteristics of the sample in more detail.

**TABLE 1:** Sample characteristics.

**Variable**	**Variable level**	**Frequency%**
Age range	20–30 years	4
	30–40 years	46
	40–50 years	50
Origin	South America	47
	Eastern Europe	46
	Africa	7
Years of occupation	<5 years	4
	5–10 years	40
	>10 years	56

All the participants have papers for a residence permit. Thus, the association through which we contacted the participants helps with the regularization of documents before helping the person to find employment.

### Measure

A semi-structured interview was developed with the aim of exploring home-care workers’ perceptions of their profession, along with the necessary skill set, with a particular focus on relationship and emotional skills (Table 2).

**TABLE 2:** Interview track.

**Thematic hub**	**Questions**
Professional story	1. Tell me about yourself and your past home-care worker experiences.
	2. Why did you choose this job?
Representation of the role	3. In your opinion, who is the home-care worker?
	4. What does it mean for you being a home-care worker?
	5. Choose an image to describe the home-care worker?
Caregiving functions and practice challenges	6. What does a home-care worker do?
	7. Describe the relationship you formed with the elderly
	8. Describe the relationship you formed with the elderly’s family.
	9. What are the challenges of your job?
	10. What would help you to overcome them?
Skill set	11. What are the necessary skills for home-care workers?

### Data Analysis

We conducted a paper-and-pencil content analysis following the interpretative phenomenological approach (Smith et al., 1999; Smith and Osborn, 2003; Smith, 2004; Brocki and Wearden, 2006), as our goal was to understand experiences and explore the meaning construction processes of professionals, while considering the relevant sociocultural context as part of the data interpretation process.

We applied a bottom-up approach to a hierarchical categorization system. Following Hsieh and Shannon (2005), we chose “conventional content analysis” (categories and names for categories flow from the data).

Following the track of data analysis proposed by Braun and Clarke (2006), first we transcribed the interviews that were previously recorded, then we reread them several times to familiarize ourselves with the content (Familiarizing with data). In a second phase, we identified the codes (Generating initial codes). We then grouped the traced codes into broader themes that could be linked to practices of the profession as well as relational and emotional challenges linked to the representation of the role (Searching for themes). Once the themes were traced, we connected the various thematic areas that emerged, creating a conceptual map. The themes were interpreted in light of the entire corpus of data (Reviewing themes). At this point, we gave a definition and name to each theme (Defining and naming themes).

Once the themes had been identified, in order to define the skill set required for home-care workers, We carried out a process of deduction and connected each theme to skills that could be useful in order to tackle that challenge or to implement a specific practice.

Data analysis was conducted by three independent coders (the agreement for each of the below-mentioned pairs of judges–CD and DG; CD and CC; DG and CC–was calculated, and after that we calculated the mean value). Using ComKappa software we verified the goodness (Cohen’s *K* = 85%) of the inter-rater reliability (Cohen, 1960; Robinson and Bakeman, 1998). Cases of disagreement were considered and discussed until consensus was reached.

## Results

The data collected revolve around two interconnected thematic hubs:

(i) Ambivalent experiences of idealization and devaluation of the profession
(ii) Uncertain professional competences

The data reveal a picture of a profession characterized by ambivalent experiences. If, on the one hand, some interviews highlighted positive aspects of this profession, underlining the components of loving care and motherly care toward the elder, on the other hand there were a large number of narratives that report a prevalence of negative and humiliating aspects of the job. Both these narratives may imply low critical and reflective capacity with regard to the role of home-care workers. It appears that choosing this profession is often the result of a forced choice dictated by economic needs. The motivation for pursuing this profession is therefore mainly extrinsic.

The polarization and inability to fully process these experiences translate to an inability on the part of professionals to mentalize and narrate the specific skill set required for this job. As part of their narration of professional practices associated with their roles, a fairly vague and uncertain picture of the role’s required competences emerges, where *hard skills* (being able to cook, clean, medicate, etc.), although clear and well defined, often overlap with specific skills tied to other professions (e.g., carrying out insulin shots, taking blood pressure measurements). Simultaneously, *soft skills* (e.g., stress management, empathy, negotiation, etc.), which might seem obvious and taken for granted, remain in the background of their narratives.

### The Role of Home-Care Workers: Between Idealization and Devaluation

As anticipated, the experiences around the profession gather along a continuum that includes, at extreme ends, the themes of idealization and social devaluation.

In fact, their narrations polarize within these two themes. If, on the one hand, some interviewees highlight the positive components of this profession, underlining motherly care aspects toward the elder, on the other hand there are some narrations that have the negative and devaluing aspects of the job as a prevailing theme.

In the stories in which idealization prevails, the interviewees talk about the care, love, and affection components that connote their jobs.

In these, the interviewees struggle to have a wider perspective on their job, mainly focusing on the positive and ideal aspects of their experience.
Interviewer: What do you think being a home-care worker means?
Interviewee: Assisting the elderly with love, what does this mean? [the interviewee was very surprised by this question and could not understand its meaning] (.) Everyone was affected by me, I also cried. We caress, they told me stories, things, secrets…

Other narrations, on the other hand, highlight the self-deprecating experience and the lack of economic and social recognition associated with the job. These themes are accompanied by a lack of contractually defined safeguarding when the workers have to independently manage their relationship with their employer.

An image to describe what the home-care worker is? (A person) who struggles to work and works for his family members who must help.
You work like a slave, that’s hard for us. Lifting a fat person with a cut leg is difficult.
You have to explain to people that we are not slaves, that we are people too, nothing else. They must understand that we should work 10 h and no more. Mentally we do, physically we do and no more.

Social devaluation is such that it induces the interviewees to interiorize this perception, as emerges from the following extract.

Interviewer: How did you come to start this profession?
Interviewee: What? Is this a profession? [ironic tone].

Specifically, the interviewee continuously reported belittling experiences linked to the lack of recognition of the profession, with strong implications regarding their self-image. Social devaluation becomes a part of a narration of the self, putting the workers at risk of a identity-personal point of view.

You feel little, because you feel that you are worthless. You are asked, “Ah, so you clean butts?”

### The Struggle of a “Second Choice”

From the encounter with family workers, a category that emerges across all interviews relates to “motivations” rooted in the choice of undertaking this type of profession.

In a majority of cases the decision to work as a family worker is determined by the need to survive in a foreign country or as a second best to the profession for which one studied, as their qualifications are not recognized. In the cases in which personal aspirations were of a different nature, the journey to this type of profession is very often accompanied by experiences of struggle, frustration, and self-depreciation. Only in a few cases, in fact, is the choice of becoming a family worker linked to the real desire of the person to embark on a professional journey in contact with the elderly, their fragility and their families–or indeed their interest in doing so.

I have a degree in economics but here it is not recognized and they made me work as a cashier.
I really like it. I did the course here in Italy to become OSS but I chose to work as a home-care worker because in RSA I could not devote all the time I wanted to my elderly, RSA have too fast dynamics. As a home-care worker I can take care of them with the calm they need.

This topic is also linked to another category that has to do with the “reasons for migration” of the individual. Indeed, there are very few cases in which the act of leaving the homeland was linked to the person’s desire; on most occasions people found themselves forced to make this decision due to the precarious circumstances they found themselves in as explained in this interview.

I didn’t choose to be a home-care worker. We couldn’t get to the end of the month, my daughter couldn’t help me. I am alone, 42 years old, I got my daughter into university. I raised my nephew. I worked in commerce. After the revolution everything changed, they privatized, I couldn’t work.

In this second scenario this emotional experience becomes key to this non-decision as it can either contribute to the trauma or migration or it can be seen as an opportunity to revive and redesign one’s life. The possibility of re-elaborating one’s own migration history can also lead to a calm professional experience as a family worker. The interviews highlight the highly traumatic experience of this event. This is accompanied by a difficulty in re-elaborating the migration journey that results in being connoted by depressing and ambivalent feelings.

It is very hard [the interviewee refers to the period when she had just arrived in Italy], I never stopped crying because I could not go back.
My life ended when I left my country. I am not living now.

A potential protective factor that emerges from the interviews in regard to this theme is represented by the relational network (social and professional) in which the person finds themself and that builds with time. Some interviews highlight the beneficial role that the positive relationship with the families of the elderly that were being assisted had.

When I go home [the interviewee refers to the country of origin], I miss Italy afterward. At home [the interviewee refers to the country of origin] now, I have no one, my daughter lives in London with my granddaughter because she studies computer science. (.) I found myself well in Italy, I always found good families. I felt good, they felt good.

Moreover, according to some family workers, another relevant aspect is linked to the impact of their home country culture on the profession. For those people coming from countries–such as South America–whose culture is characterized by great value placed on the elderly in families and the community, the passage to a profession that doesn’t necessarily represent a voluntary decision is less hard and can be more easily integrated coherently in one’s own perceived identity.

Because, you know. there are those like us [South Americans] for whom the family and the elderly are important. And now I understood many things about what my mom did for my grandparents that I didn’t understand when I was a child. They are memory, history, tradition, our roots. But then there are those, like Eastern Europeans. those not… eh. I have seen it many times. it is not that they [Eastern Europeans] are bad. But they are more abrupt. that is, sometimes they [Eastern Europeans] also have more anger.

### A Skill Set That Is Still Uncertain

The data collected highlight difficulties on the part of interviewees in focusing on and verbalizing the specific skill set needed in this profession.

This can be interpreted in light of the lack of formalized training programs associated with the job. At the same time, the lack of boundaries of the skill set can also be interpreted in light of the self-deprecating or idealizing emotional experiences that characterize the profession.

#### The Need for Hard Skills

On one hand, the “hard” skill set component is clearly indicated, linked to the management of the home or maternal care of the elderly. The hard components, however, overlap with and are confused with skills linked to other professional profiles like social health workers or nurses, as reflected in this interview:

You have to clean, clean the house, prepare food, take pressure, prepare insulin.

This raises questions about the professional boundaries of the labor profile of the home-care worker that seem to incorrectly intersect with other more institutionalized professions.

The area of emotional and relational skills linked to this profession also remains open and not well defined.

### Qualitative Engagement of the Elder

Firstly, it can be seen that the time that family collaborators spend with an elderly person that they assist not only engages them “quantitatively” but also “qualitatively,” Thus, it is clear how important it is to know the tastes, routines, and personality nuances of the elder in order to qualitatively improve the management of their daily life. One of the interviews provides the following example:

When I saw my lady, when I saw her a little down, I always gave her a chocolate because she liked them so much and she immediately gave me a big smile. Or I’d put on her songs, sing them and make her dance.

This extract suggests the need to strengthen contextual skills relative to being able to adequately read and interpret the domestic family setting one finds oneself in, in order to efficiently fulfill the needs of the elder.

### The Solitude of Routine

There are also a series of emotional challenges that emerge that are linked to living in close contact with an elderly person who is ill. This results in lived experiences of fear, anxiety, and stress, especially when the elderly person exhibits behaviors due to their illness that can be harmful for themself or others. At the same time, another emerging theme is the solitude of professionals in managing this emotional burden both during their work and also when their contract ends due to the death of the elder.

I went to this lady. She was very nervous, with dementia. We slept together. I was afraid and I slept with her in bed because she always said that her neighbor comes and steals. It was very difficult.
When they die they slowly take away a piece of my heart.
We have so many things inside that we can’t tell anyone, we have so many difficulties, you can’t say anything.
Most of mine have Alzheimer’s, dementia, they’re nervous, other times they don’t want to wash. I insist. This last one… I am fond of her, I cried like I cried after my father’s [death].

These challenges suggest the need to create a profile of desired personal skills for these roles, such as stress and anxiety management.

### The Relationship With the Elder’s Family

Finally, the interviews highlight challenges linked to the relationship with the relatives of the assisted as reflected by this interview.

Sons have trust in us. The daughter didn’t accept the condition of the mother. Doctors said that the mother should stay in bed but she wanted me to clean her in the bath. I fell down several times because of it. It was difficult for her to believe that her mother was at that point. She didn’t trust me, she called me several times every day. (.) I thought that she was jealous of me.

In particular, it is apparent that conflict can characterize these relationships. The psycho-physical condition of the elder can be difficult to accept for sons and/or daughters. In this sense, we can see a projective dynamic in which the son/daughter unconsciously applies his/her frustration, helplessness, and anger regarding the elder’s position to their relationship with the home-care worker. On the other hand, home-care workers find themselves powerless in these tense situations with the result that they live them with anger and helplessness, like the family, with no way of finding a solution.

It is evident that there is a need to train workers in developing relational skills linked to empathy, mediation, and negotiation of conflict, which can be useful in better dealing with these circumstances.

### Potential Dysfunctional Working Relationships

The precarity of the profession can expose professionals to multiple traumas linked to dysfunctional working relationships that, in some cases, end up damaging the dignity and rights of the person.

Once I left because the man touched me (.).
Three months ago I had to do a job; I worked 13 h instead of 10 as stated in the contract. I have not seen the amount due to me. The guy began to shout, “Stupid! When I get pissed I scream.” So, sir, I’m leaving, I have my pride. When they shout at me I can’t stand it. I do work, I do too much work. I do everything 100%. It was written, I have seen the contracts.

In this sense, the mediation of a recruiting agency could guarantee better safeguarding of professionals, who should therefore feel less alone when facing possible harassment or injustice from their employers.

## Discussion

The home-care worker profession is a profession with boundaries that are still blurred and uncertain, not only for outside observers but also for the professionals themselves. The job of the home-care worker, in fact, inevitably implies a mixture of different skills, from performance-related skills to emotional and relational skills between multiple parties. Looking at the workers’ perspective, the motivations for carrying out this profession are intertwined with personal stories that brought the workers to Italy and with the circumstances they found themselves in once they arrived in our country. The choice of this profession is primarily driven by economic needs. If we shift the interpretation of these data on a level linked to the role representations, it is clear that in cases where the motivation is extrinsic, the interpretation of the role will tend to fall into line with the prescriptive dimension only, at the expense of the discretionary one. Therefore, there is a risk that this results in an incompatible division between the “personal ideal identity” and the “real professional one.” In addition to this aspect, in many cases there is also the element of necessity, for which this type of profession represents the only means of survival. Moreover, according to home-care workers’ representations, this role is very often characterized by a deep professional solitude, which sometimes is also personal. It becomes important to then build an assistance and support network that allows them to find opportunities for discussion, to capitalize on the know-how built with experience, and build awareness of the limits and boundaries to safeguard themselves as professionals. Indeed, it is rare that in their narrations the professionals are able to translate their experience into competences, despite–as can be seen from the interviews–the job of a home-care worker being one that requires a wide range of skills. From their stories and from their representations of their role, it appears that this profession is one that an individual finds themself in where one improvises, finding one’s own measure based on previous experiences and on account of one’s personal inclinations. Having come across a common difficulty not only in giving a name to but also in recognizing on a conscious level how to recognize competences, it seemed important, through an *ex post* inference operation, to reread the interviews and outline a group of necessary competences (Table 3) in light of the activities required by this profession, taking care not to be reductive in terms of the psycho-emotional implications of the job.

**TABLE 3:** Snapshot of the skill set deduced from repertoires of practices and caretaking actions required from home-care workers.

**Soft skills**	
Personal skills	–Creativity
	–Flexibility
	–Self-awareness (limitations and resources)
	–Precision and punctuality
	–Stress management
Interpersonal skills	–Motherly care
	–Empathy
	–Involvement of relatives
	–Mediation
	–Negotiation
Contextual skills	–Observation of needs and routine of the assisted
	–Observation of family and domestic dynamics
	–Care in the passing of information
Domestic management skills	–Cooking
	–Cleaning
	–Looking after the space
**Hard skills**	
	–Hygene of the assisted
	–Help in the carrying out of physiological functions
	–Ensuring appropriate nutrition
	–Mobilization and help in deambulation
	–Maintaining residual psycho-physical functions and skills
	–Administration of medicine
	–Performing simple health-care tasks (*if with a social health worker diploma*]

In terms of soft skills, on a personal level this work profile requires being creative and flexible in order to adapt to different circumstances. Creativity in this context is not intended in its strict definition in terms of the process of creation of new and useful ideas with the intent to innovate (Amabile, 1983), but rather, this refers to knowing how to be able to take advantage of the circumstances one finds oneself in, the limitations (for example, the impossibility of leaving the domestic environment) and the available resources (for example, musical entertainment) to find effective solutions that enable the containment of difficulty in managing a challenging situation.

A counterpart to the two previous skills is being able to adopt a certain rigor in terms of precision and punctuality in order to better preserve the elder’s living conditions and guarantee a serene and stable living environment. Also, in this case it might be useful to clarify that rigor, precision, and punctuality do not mean having a rigid and uncompromising approach, but rather, this is intended as a precaution in the creation and respect of routines relating to the elder’s rhythms. We are then talking in terms of programming and coordinating the various phases of the day, while bearing in mind the respective peculiarities and possible setbacks.

Naturally, having to find a balance between these different dimensions, it is crucial to have good self-awareness as well as a knowledge of what one’s inclinations are in order to counterbalance the areas with more deficiencies. Relational skills also play a key role in maintaining the elder’s emotional well-being. Motherly care and empathy–meaning attunement with needs of various natures of the elder and coherent caregiving responses and actions–are certainly necessary but not enough. Having to work in constant contact with the private world of the elder, it is also necessary to be attentive in involving the relatives and, in the majority of cases, knowing how to mediate and negotiate different opinions, expectations, and demands. All of this is particularly difficult and delicate as the risk becomes that of (unconsciously) falling into a triangulation dynamic (Bowen, 1985) in which the home-care worker can be placed in a “third party” position toward or through whom to move tensions and conflicts within the family system. A first key step consists of training focused on developing awareness of these risks and the management of relational and communication dynamics in order to set boundaries that are acceptable for all parties.

Contextual skills can also be helpful. Being able to respond to the needs and habits of the assisted person, for starters, can facilitate the integration of the home-care worker within the family system and can provide him/her with the most relevant and useful information for the management of the elder’s daily life.

Moreover, being able to perceive the dynamics that characterize a specific family nucleus can facilitate the home-care worker’s work not only in communicative exchanges and in the sharing of information, but also in the understanding and interpretation of events as they occur. Being capable of naming what is being staged can be a precious compass in governing contact between two parties that are strangers to one another (family and home-care worker) and provide an emotional thermometer to support the home-care worker in positioning herself in an effective and protective way.

Finally, in the soft skills domain, some domestic management skills have been included as, although they have to do with cleaning, cooking, and looking after the space, in reality domestic management implies the respect of the private and intimate world of the elder–a key factor when linked to all the competences that have been previously discussed.

In terms of hard skills, a series of skills that deal with the physical and mental well-being of the elderly have been included, such as personal hygiene, ensuring adequate nutrition, mobilization and help in walking, as well as finding strategies and activities that allow for the maintenance of basic functions and psycho-physical skills. Despite the current absence of obligatory training programs and the lack of a formal issued qualification, these types of skills cannot be taken for granted or automatically acquired on the job. Practice certainly helps in consolidating these skills, but a basic and “safe” level of training needs to be considered (D’Angelo et al., 2020).

The issue of giving medicine and performing simple medical treatments deserves a side reflection. If for the administration of therapies it can “be enough” to build a trusting and delegating relationship with the relatives, the subject of medications cannot not be equally simplified. This type of competence, in fact, is required by a different professional profile. It is therefore very important to clarify what the boundary is and the dynamics with other social welfare figures (e.g., social health workers), and to ask what the boundaries and responsibilities that a home-care worker can legitimately take are.

A key role in this sense can be played by realities that link family collaborators with families, highlighting the most coherent professional profile based on the family’s specific demands.

## Conclusion

As shown by demographic data and literature, the growing demand for home-care workers requires both research and practice to reflect deeply on this issue (Hall and Coyte, 2001; McClimont et al., 2004; Broese van Groenou et al., 2006; Stone and Dawson, 2008).

A first theme in the role’s representation is linked to a significant gap between the effective measure of value of this profession and the recognized value given to it. This gap is not only associated with “Invisible Welfare” and support for families, but also with making sense and meaning for whoever carries out this job.

In the majority of cases, home-care workers themselves talk about a profession that is perceived by others, and experienced by them, as a job that one “finds oneself doing” without any other choice (or for survival), a fallback in respect to what they would have wanted to do and, due to this, they struggle to see their experience as developing skills. In addition to this, there is an idealized conceptualization–typical in aid professions–according to which personal inclinations, based on goodness of heart and the desire to help others, automatically become the bearers of skills and performance efficacy, implicitly making dimensions such as struggle and conflict incoherent.

The study confirms that the professional profile of the home-care worker still remains poorly defined and that the professionals themselves struggle to articulate what the skill set they develop comprises.

This is not linked to putting a label on a skill and “naming things,” but rather promoting a process of acknowledgment, recognition, and valorization of the invisibility that permeates this profession.

In this regard, the research highlights the fatigue of home-care workers in making sense retrospectively of their professional experience (Heldal and Tjora, 2009). As reported by O’Connell (2003), making sense implies reaching an understanding that was not clear before. However, being a retrospective process (that is applicable to events that have already happened), for home-care workers it becomes a step not to be taken for granted because they don’t always have the tools, space, and time to interpret their professional experience (Weick et al., 2005).

The interviews suggest that, apart from practical skills linked to management of the house and the material care of the elder, there is another area of skills–the so-called “gray area”–whose boundaries are not yet clearly recognized and shared. In this area we can trace those skills that are more linked to the immaterial care of the elder, dynamics that are usually classified as humane and referred to with terms such as “honesty,” “patience,” “reliability,” “joyfulness,” and “gentleness.” Therefore, there is awareness that the psycho-emotional component plays a key role; however, we can affirm that there is still a lack of a shared vocabulary to define not only in a more precise and accurate way but also in a way that is more shared. Many studies have already highlighted the fragility and risks of this profession, which are linked to the large emotional and relational load that prolonged contact with the elder person carries and the importance of the relationship with relatives (Näre, 2011; Sardadvar et al., 2012; Triandafyllidou and Marchetti, 2015). These risk factors represent challenges that are intrinsic to this profession and are somehow an integral part of its implementation. As a result, all home-care workers are exposed to the psycho-emotional risk of being in a close relationship with the elder and with the relatives. In light of these risks and challenges, it is important to train them through training programs that can help, through the development of adequate skills, to manage the complexity of this profession and safeguard not only the psycho-physical well-being of the assisted person but also one’s own well-being as well.

The creation of training programs curated by, for example, the non-profit sector or government bodies, moreover, could have a significant impact on two issues that hold particular social relevance. On the one hand, doing so will help reduce the high level of informal work taking place in this profession, which is estimated to be 90% in total. On the other hand, it will promote the definition of working contracts tailored to competences that are pertinent to the required profile, guaranteeing working conditions that are protective and reducing the risk of demands for non-coherent tasks. With regard to the contract issue, it is also worth underlining that in the definition of the contract the presence of a third party–such as, for example, an association–represents a protective factor that not only guarantees the quality of service, but also protects the professionals and their rights.

Talking about safeguarded working conditions and on-the-job demands not only refers to hard skills (for example, administering simple medication without possessing a diploma) but also to the set of soft skills that, as repeatedly mentioned, is often discarded and not valued.

A first area of crucial competence concerns personal skills such as creativity, flexibility, self-awareness (limitations and resources), precision and punctuality, and stress management. This area of skills could not only help in effectively managing daily life, but also by spicing up the monotony and repetitiveness of some aspects of the job, especially when one has to handle by oneself the routine of an older person with cognitive and/or physical difficulties. At the same time, the consolidation of skills to manage stress can help in emotional overload moments, which, as demonstrated, are a part of this profession (Näre, 2011; Sardadvar et al., 2012; Triandafyllidou and Marchetti, 2015). This could also help avoid an identity conflict in these professionals that, as reported by this study, suggests they embarked on a journey in this profession because they “needed to” (extrinsic motivation) rather than because it was a “vocation” (intrinsic motivation). It is important to help the home-care worker in consolidating an adequate level of self-awareness in order to recognize and find value in the profession, and give meaning to their history by softening the difference between his/her personal identity and his/her ideal identity.

It is also important to support home-care workers in adequately managing their relationships, due to the fact that the relationships with the elder person and his/her relatives constitute a crucial node of the profession. This means supporting them in terms of motherly care, empathy, the involvement of relatives, mediation, and negotiation.

On the one hand, the skills of motherly care and empathy are key as they allow for the addressing of the family and elder’s need at a time that is so delicate and painful in the cycle of family life (Cigoli and Scabini, 2006). On the other hand, especially in regard to mediation and negotiation skills, these skills can be useful to the home-care worker for preserving and maintaining professional and relational boundaries and therefore safeguarding his/her psycho-physical wellbeing. The negotiation of breaks, working hours, or days off is a key and emblematic aspect of this process.

Contextual skills–the observation of needs and habits of the elder; the observation of domestic dynamics; care in the sharing of information–are just as crucial as they allow the worker to move with sensitivity and mastery within this context, allowing for the recognition of key signals and ones that confirm the efficacy of the service. These skills are also strongly linked to those concerning domestic management, intended not merely as material tasks, but as a further means of expression of attention to the relationship and a caretaking dimension of the emotional and identity domain of the elder.

## Limitation

Although of an exploratory nature, our contribution was the first attempt at profiling the skill set of the home-care worker profession. In light of these first findings and the exponential demand that will characterize this profession, future studies with a bigger and more heterogeneous sample size are warranted. It would also be interesting to look into the relatives’ perspective of this profession and the skill set needed for it. In sum, we can conclude by stating that, although the profession of the home-care worker has many risks and challenging elements to it, it is a profession rich in nuances. For this precise reason, it is important to focus on workers’ awareness of the value and the resources that can be put in place to avoid a dangerous overexposure in terms of demand.

## Data Availability Statement

The raw data supporting the conclusions of this article will be made available by the authors, without undue reservation.

## Ethics Statement

The studies involving human participants were reviewed and approved by Commissione Etica per la Ricerca in Psicologia CERPS–Università Cattolica del Sacro Cuore. The patients/participants provided their written informed consent to participate in this study.

## Author Contributions

All authors worked together on the research project and worked on the data analysis and interpretation. DG and CC worked on the data collection.

## Conflict of Interest

The authors declare that the research was conducted in the absence of any commercial or financial relationships that could be construed as a potential conflict of interest.

## Footnotes

1 In Italian, home-care workers are usually called “badanti.”

2 https://professionioccupazione.isfol.it/index.php?lang=en&top=1&testo_percorso=&link_percorso=&testo_subpercorso=&link_subpercorso=&id_menu_principale=1&id_categoria=0&div=white&testo_percorso=&link_percorso=&testo_subpercorso=&link_subpercorso=
